# Empowerment through agency enhancement

**DOI:** 10.3389/fpsyg.2022.1060256

**Published:** 2022-11-03

**Authors:** Zhiyue Tong, Fengcun An

**Affiliations:** School of Foreign Languages, Yanbian University, Yanji, China

**Keywords:** agency, enhancement, development, marginalized communities, interdisciplinary exploration

Agency has received considerable attention during the last decade and is gaining increasing popularity in the exploitation of psychology field. As Antti et al. (2016) state, “Agency is hence closely related to autonomy and power relations in human activity and learning” (p. 1). Numerous scholars have defined agency as a psychological term. For instance, Gao (2010) indicates that agency is commonly defined as an individual's will and capacity to act, which reveals the centrality of agency to learner identity, learner autonomy, and learner behavior in particular and sheds a light on the importance of empowering learners to lead their own learning. Previous studies have moved agency research forward in several respects. However, despite some researchers' attempts to facilitate such processes, those studies have encountered substantial challenges. The book *Empowerment through Agency Enhancement* written by Mine Sato, Nobuo Sayanagi, and Toru Yanagihara theoretically and practically addresses these challenges and the research gap and provides a blueprint for further agency research.

The book consists of three main parts, each including three chapters, besides the introductory and concluding sections. Part I (Chapters 2–4) discusses how each discipline defines agency and describes agency development from a diachronic perspective. Part II (Chapters 5–7) focuses on enhancing agency and explores plausible hypotheses and the theoretical basis of agency development while discussing the enhancing and thwarting factors. Part III (Chapters 8–10) illustrates specifically how to make processes of agency development *visible* by verbalizing, patterning, and devising frameworks for evaluations.

Chapter 1, Introduction, outlines the book and the researchers' motivation for taking the initiative to conduct this interdisciplinary research. Chapter 2 discusses how culture is defined and seeks to uncover a hidden connection between culture and agency as well as agency development. Chapter 3 provides a selective review of the psychological perspective of agency in terms of the psychological construct and brief history. This chapter also concentrates on the relationships between self-efficacy, self-determination theory (SDT), and agency. Chapter 4 contextualizes the definition of agency in traditional economic literature and presents the exposition in three distinct contexts.

Chapter 5 elaborates the plausible dynamics of agency development in practice through relevant academic theories and development projects with a case analysis led by an unsung expert to propose a hypothesis for enhancing agency in practice. Chapter 6 puts forward another hypothesis, a modification of SDT, which is considered the central mechanism of agency, especially for marginalized populations to ameliorate autonomous motivation. This chapter also concentrates on poverty reduction among beneficiaries through developing aid projects, which can influence other populations as well. Chapter 7 addresses issues surrounding the user-centered approach (UCA) to service provision, which regards agency as an outstanding feature of service users.

Chapter 8 presents historical elaborations of the Life Record Movement (LRM) in the post-war context, exploring effective suggestions in the current situation and international projects that involve agency development, self-reliance, and empowerment components. The penultimate chapter (Chapter 9) proposes the underlying assumptions of the psychometric model and probes into the difficulties of measuring agency and autonomous motivation in the poverty context. Chapter 10 starts with an in-depth case study concerning the Chile Solidario Program (CHS) proposed and implemented by the national government of Chile to offer assistance for the poorest segment of the population to move out of poverty. Finally, the concluding chapter dissects all the preceding chapters to present differences and similarities concerning definitions, mechanisms, and visualizations of agency and its development.

This monograph contributes to evolving research aimed at enhancing and visualizing individuals or marginalized populations in three related contexts—namely, participatory development, extension work, and service transactions. It also reflects the depth and breadth of research on agency enhancement, both theoretically and methodologically, in terms of factors and mechanisms. Theoretically, this book not only delineates a theoretical road map for the definition of agency but also displays how each theory can be adopted to facilitate and activate agency in practice. Drawing from theoretical frameworks concerning self-efficacy, SDT, and UCA, the book sheds light on the critical role played by the agency and offers insight to provide readers with a general understanding of agency enhancement by focusing on volition and promoting *eudaimonia*.

Methodologically, one outstanding feature of the book is that it provides explanations from multiple perspectives and attempts interdisciplinary approaches, such as anthropology, economics, and psychology, to identify and formulate agency systematically. The authors manage to draw insights, suggestions, and implications for the practice of agency enhancement aimed at enabling or engaging people to take initiatives across disciplines. As mentioned above, the psychology parts provide direct, clear, and useful guidance and tools for implementing the practice of agency development in terms of measuring the degree of autonomy in the motivation. By comparison, the anthropology and economics chapters offer some indirect characteristics. The authors shed light on the nature of the profound change involved in the process of realizing “power from within” and focus on the person-specific factor in the anthropology chapters. The economics chapters provide useful conceptual frameworks for visualizing the dynamic process of agency development and the distinctive modes of engagement by workers at different stages of the process. The other outstanding feature is that the authors employ a mixed-methods approach to study agency, which involves quantitative measurements and qualitative data, and it is necessary to solidify the foundations of psychological studies, especially studies on marginalized populations in poverty contexts. Psychological researchers will be enlightened by the book and benefit tremendously from its systematic identification of the factors and mechanisms.

With a thoughtful concern regarding the aforementioned issue, the authors conduct this research on agency and its development from interdisciplinary perspectives with a combination of quantitative measurements and qualitative statistics to fill the gaps. More concretely, the authors design the research project to enhance the agency of marginalized communities, which has become the key to empower them through bridging practices and theories and applying bottom-up development. Moreover, this book provides readers with a deeper discussion about empowerment which is an activation or enhancement of agency in human capabilities when various types of anxiety emerged among the marginalized. To sum up, all the praiseworthy features above make the book enlightening for the front-line individual in response to empowerment and agency enhancement, especially in the context of the post-pandemic era.

This book has opened an agenda of agency enhancement to fill the gaps for the marginalized and can be a valuable source for researchers and instructors who show great interest in analyzing students' agency systematically and are intended to enhance learners' agency in various educational contexts. Despite its slightly vague illustration of some plausible approaches such as “User-Centered Approach” and measurement of visualized stages of agency development in practice, the monograph, in general, provides multiple approaches and a new measurement of agency as a means of empowerment. Even though the authors attempt to probe the issue of agency from interdisciplinary perspectives, it would be more comprehensive and holistic if the authors can form a network with other interdisciplinary research teams in the related fields. This, in fact, may leave a research space for further exploration.

## Author contributions

ZT drafted the general commentary. FA revised the text and provided some critical and constructive opinions to the draft. All authors listed have made a substantial, direct, and intellectual contribution to the work and approved it for publication.

## Funding

This review was supported by the National Philosophical and Social Project entitled The Construction of Languages Capacity in Border Areas of China [20BY067Y].

## Conflict of interest

The authors declare that the research was conducted in the absence of any commercial or financial relationships that could be construed as a potential conflict of interest.

## Publisher's note

All claims expressed in this article are solely those of the authors and do not necessarily represent those of their affiliated organizations, or those of the publisher, the editors and the reviewers. Any product that may be evaluated in this article, or claim that may be made by its manufacturer, is not guaranteed or endorsed by the publisher.
